# A Complicated Case of Aspirin-Exacerbated Respiratory Disease With Kounis Syndrome

**DOI:** 10.7759/cureus.45635

**Published:** 2023-09-20

**Authors:** Krista M Shaw, Brittanie I Neaves, Hayden A Springer, Christopher A Coop

**Affiliations:** 1 Internal Medicine, Keesler Medical Center, Biloxi, USA; 2 Allergy and Immunology, Keesler Medical Center, Biloxi, USA

**Keywords:** allergic myocardial infarction, allergic acute coronary syndrome, aspirin hypersensitivity, aerd, aspirin-exacerbated respiratory disease, kounis syndrome

## Abstract

Kounis syndrome is angina or acute coronary syndrome caused by mast cell degranulation and inflammatory cell activation. We present a case of a patient with underlying aspirin-exacerbated respiratory disease (AERD) and previous anaphylaxis to aspirin. The patient underwent aspirin desensitization and was then treated with high-dose aspirin. Unfortunately, he developed recurrent angina and myocardial infarction (MI). Numerous left heart catheterizations revealed vasospasms as the etiology of his MIs; however, therapy with increasing doses of vasodilators yielded no improvement in the patient’s condition. Ultimately the patient’s aspirin was discontinued and he had no recurrence of angina or MI.

## Introduction

In Kounis syndrome, acute coronary syndromes are triggered by allergic, hypersensitivity, or anaphylactic reactions which cause activation of inflammatory cells and mediators leading to vasospasm and MI [1]. Multiple medications, foods, and environmental exposures are known to precipitate Kounis syndrome, and patients with certain underlying conditions, including asthma, are at increased risk [2]. The management of Kounis syndrome is difficult because hypersensitivity coronary syndromes may be underrecognized and because standard treatments for MI may not be appropriate for vasospasm.

## Case presentation

A 28-year-old male US military service member with chronic allergic rhinosinusitis and asthma presented with acute onset of respiratory failure immediately after taking aspirin. He was emergently intubated and treated for anaphylaxis with rapid improvement. He was seen in an allergy and immunology clinic and managed with omalizumab and fluticasone/salmeterol for his asthma. His allergic rhinitis was managed with cetirizine, montelukast, and immunotherapy. He was counseled on aspirin and other non-steroidal anti-inflammatory drug avoidance and provided with intramuscular epinephrine autoinjector in case of anaphylaxis. The patient was then temporarily lost to follow-up due to military deployments.

Four years later, the patient reported worsening rhinosinusitis. He underwent functional endoscopic sinus surgery/polypectomies and aspirin desensitization to reduce further nasal polyp formation. The two-day aspirin desensitization protocol described by Lee and Stevenson was followed, and the patient received increasing doses of aspirin while being monitored with hourly spirometry [3]. He experienced transient chest tightness and a 10% decrease in forced expiratory volume in one second (FEV1); however, he was able to complete the protocol. He was discharged home on aspirin 650 mg twice daily which was tapered to 325 mg daily. The patient initially reported significant improvement in his quality of life with well-controlled asthma and with minimal return of nasal polyps.

**Figure 1:**
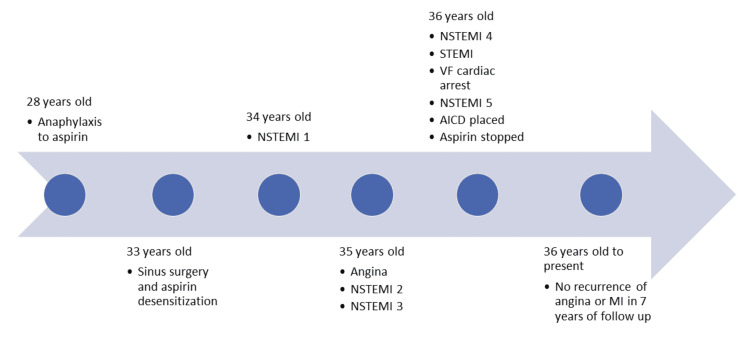
A timeline of the patient’s aspirin-associated respiratory and cardiac events. NSTEMI: non-ST-elevation myocardial infarction; STEMI: ST-elevation myocardial infarction; VF: ventricular fibrillation; AICD: automatic implantable cardioverter defibrillator; MI: myocardial infarction

One year later, the patient presented with crushing substernal chest pain and troponin I elevation to a peak of 3.6 ng/mL (0.020-0.050 ng/mL). His EKG was unremarkable. He underwent a left heart catheterization and was noted to have multiple high-grade lesions in his coronary arteries. A stent was placed in his posterior descending artery. Interestingly, the other lesions were found to be completely resolved. This was thought to be most consistent with vasospasm. The patient was treated with dual anti-platelet therapy due to stent placement; clopidogrel was started and his previously prescribed aspirin 325 mg daily was continued. He was discharged on these medications as well as on diltiazem and nitroglycerin for the prevention of vasospasm. Over the next two years, the patient had multiple admissions at different hospitals for episodes of chest pain and MI (Table 1 and Figure 1).

**Table 1:** Multiple hospitalizations for angina and myocardial infarction. NSTEMI: non-ST-elevation myocardial infarction; PDA: posterior descending artery; RCA: right coronary artery; LCx: left circumflex artery; STEMI: ST-elevation myocardial infarction

Facility	Patient presentation	Left heart catheterization findings	Troponin I level (laboratory-specific normal troponin I range)
1	NSTEMI	Diffuse coronary lesions. Stent placed in PDA; other coronary lesions resolved spontaneously, consistent with spasm	3.608 ng/mL (0.020-0.050 ng/mL)
2	Angina	Spasm of RCA	Not available
3	NSTEMI	Spasm of RCA and LCx	0.30 ng/mL (0.00-0.03 ng/mL)
3	NSTEMI	Not performed	0.573 ng/mL (0.00-0.301 ng/mL)
4	NSTEMI	Patent coronary arteries	1.24 ng/mL (0.00-0.03 ng/mL)
5	STEMI	Diffuse coronary spasm	Not available
3	NSTEMI	Not performed	1.42 ng/mL (0.00-0.03 ng/mL)

He underwent numerous cardiac catheterizations showing vasospasm. Urine toxicology screening was repeatedly negative. His doses of calcium channel blockers and nitrates were increased and ranolazine was started without an improvement in his symptoms.

Finally, the patient had an ST-segment elevation MI. Left heart catheterization showed diffuse coronary artery spasm. The procedure was complicated by two ventricular fibrillation cardiac arrests, each with immediate successful defibrillation to normal sinus rhythm. An automated intracardiac defibrillator was then placed for secondary prevention of sudden cardiac death.

At that time it was determined that all of the patient’s MIs had occurred while he was being treated with aspirin 325 mg daily. The aspirin was discontinued, and the patient has now been followed for several more years with no recurrence of chest pain or MI. The patient’s rhinosinusitis has been managed with dupilumab and the occasional polypectomy.

## Discussion

Aspirin-exacerbated respiratory disease (AERD) comprises a triad of rhinosinusitis with nasal polyps, asthma, and intolerance to aspirin and non-steroidal anti-inflammatory drugs. Aspirin intolerance may manifest as upper airway symptoms, such as rhinorrhea, or as lower airway symptoms, such as bronchoconstriction and laryngospasm [4]. While aspirin hypersensitivity in AERD is not IgE-mediated, reactions to aspirin may mimic IgE-mediated anaphylaxis [5]. The pathophysiology of AERD is not fully understood; however, it is known to involve the activation of eosinophils and mast cells and increased production of cysteinyl leukotrienes. COX-1 inhibition leads to decreased prostaglandin E2 synthesis and increased activation of mast cells and eosinophils [5,6]. This in turn causes increased production of histamine, tryptase, and leukotrienes, leading to bronchospasm and increased mucus production [5]. Dupilumab, which increases prostaglandin E2 and decreases leukotrienes, results in significant improvements in both upper and lower airway symptoms [6].

Initial therapy in AERD involves corticosteroids, bronchodilators, and leukotriene antagonists. Often these measures are insufficient and aspirin desensitization may be performed or the patient may be treated with dupilumab or mepolizumab [6]. During aspirin desensitization, patients are provided with increasing doses of aspirin until the medication no longer induces a hypersensitivity reaction. Patients who have undergone desensitization experience decreased polyp formation and they are often protected from further respiratory reactions to aspirin and NSAIDs [3,4].

While aspirin is a potential therapy for AERD, it may also be responsible for adverse events. Aspirin has been reported to be associated with vasospasm both in patients with AERD and in those without it [7,8]. Aspirin is thought to contribute to vasospasm through disturbing the balance of prostaglandins, which favor vasodilatation, and thromboxane A2, which causes vasoconstriction [7].

In one case series, 10 patients with AERD were found to have angina due to vasospasm [8]. Of these, eight patients had undergone aspirin desensitization and were on aspirin therapy, and six of them reported that their symptoms worsened with aspirin therapy and remitted with reduced dosing or discontinuation of aspirin [8]. Other case reports also show that patients with AERD had coronary vasospasms immediately after taking aspirin or other NSAIDs [9,10].

In Kounis syndrome, allergic or hypersensitivity reactions cause acute coronary syndromes. Inciting factors of Kounis syndrome include medications including NSAIDs, foods, and environmental exposures [1,2]. The pathophysiology of Kounis syndrome involves mast cell and other inflammatory cell activation and the release of inflammatory mediators including histamine, tryptase, leukotrienes, and thromboxane, which are also implicated in AERD [2,6]. Kounis syndrome has three defined subgroups, type I involves coronary vasospasm, type II involves coronary artery plaque rupture as a result of allergic reaction, and type III is stent thrombosis in which the thrombus has eosinophils and mast cells [2].

Our patient experienced Kounis syndrome type I, triggered by aspirin, in the setting of a previous history of aspirin hypersensitivity. He was severely affected by Kounis syndrome, undergoing MIs, cardiac arrests, and automatic implantable cardioverter defibrillator (AICD) placement. The diagnosis was delayed due to the underrecognition of hypersensitivity angina. Aspirin was not initially considered as an etiology of vasospasm, it was believed that continued aspirin therapy was indicated for AERD treatment post aspirin desensitization. Additionally, after the patient received a coronary stent, aspirin was then indicated for the prevention of stent thrombosis. The patient sought care at multiple facilities due to military deployments, which likely delayed the diagnosis. Fortunately, aspirin was ultimately recognized as the cause of the patient’s coronary vasospasms, and the patient has had no angina or MI since it was discontinued.

## Conclusions

In conclusion, we presented a case of a patient with AERD who underwent aspirin desensitization, started on aspirin therapy, and then experienced recurrent angina, MIs, and cardiac arrests. Aspirin was discontinued, and the patient had no further cardiac symptoms. Aspirin therapy can be an important part of the treatment for AERD in patients who have undergone aspirin desensitization; however, aspirin therapy increases the risk of bleeding, and patients with a history of respiratory reactions to aspirin could also be predisposed to aspirin-induced vasospasm or MI. Biologic therapy with dupilumab or mepolizumab may be a safer alternative to aspirin desensitization in some patients. Kounis syndrome must be considered as the etiology in any patient with vasospastic angina or MI, especially in those with underlying hypersensitivities. In patients with AERD who develop vasospasm while on aspirin therapy, aspirin discontinuation could prevent MI, arrhythmia, and cardiac arrest.
